# The MDT-15 Subunit of Mediator Interacts with Dietary Restriction to Modulate Longevity and Fluoranthene Toxicity in *Caenorhabditis elegans*

**DOI:** 10.1371/journal.pone.0028036

**Published:** 2011-11-21

**Authors:** Jennifer Schleit, Valerie Z. Wall, Marissa Simko, Matt Kaeberlein

**Affiliations:** Department of Pathology, University of Washington, Seattle, Washington, United States of America; Brown University, United States of America

## Abstract

Dietary restriction (DR), the limitation of calorie intake while maintaining proper nutrition, has been found to extend life span and delay the onset of age-associated disease in a wide range of species. Previous studies have suggested that DR can reduce the lethality of environmental toxins. To further examine the role of DR in toxin response, we measured life spans of the nematode *Caenorhabditis elegans* treated with the mutagenic polyaromatic hydrocarbon, fluoranthene (FLA). FLA is a direct byproduct of combustion, and is one of U.S. Environmental Protection Agency's sixteen priority environmental toxins. Treatment with 5 µg/ml FLA shortened the life spans of *ad libitum* fed nematodes, and DR resulted in increased sensitivity to FLA. To determine the role of detoxifying enzymes in the toxicity of FLA, we tested nematodes with mutations in the gene encoding the MDT-15 subunit of mediator, a transcriptional coactivator that regulates genes involved in fatty acid metabolism and detoxification. Mutation of *mdt-15* increased the life span of FLA treated animals compared to wild-type animals with no difference observed between DR and *ad libitum* fed *mdt-15* animals. We also examined mutants with altered insulin-IGF-1-like signaling (IIS), which is known to modulate life span and stress resistance in *C. elegans* independently of DR. Mutation of the genes coding for the insulin-like receptor DAF-2 or the FOXO-family transcription factor DAF16 did not alter the animals' susceptibility to FLA compared to wild type. Taken together, our results suggest that certain compounds have increased toxicity when combined with a DR regimen through increased metabolic activation. This increased metabolic activation appears to be mediated through the MDT-15 transcription factor and is independent of the IIS pathway.

## Introduction

Dietary restriction (DR) has been shown to increase life span in multiple organisms including yeast, flies, nematodes, mice, and monkeys [1], [2], [3], [4], [5]. In addition to life span extension, DR has also been shown to reduce age-related disease [4], leading to the suggestion that DR or small molecules that mimic DR could be utilized to improve healthspan in people [6], [7], [8], [9]. Several pathways have been implicated in the response to DR, including increased activity of sirtuin protein deacetylases, reduced insulin/IGF-1-like signaling (IIS), and reduced activity of the target of rapamycin kinase [10], [11], [12], [13], [14].

Although there is abundant evidence that DR can increase life span and enhance healthy aging in evolutionarily divergent species, the beneficial effects of DR do not appear to be universal. For example, DR increased maximum but not median life span in one strain of wild-derived mice [15]. In a recent study of 40 recombinant inbred mouse lines, a 40% reduction in caloric intake failed to extend life span in more than half of the strains examined [16]. In addition, DR has been reported not to increase life span in several different genetic backgrounds, including yeast and mice lacking sirtuin-family proteins [17], [18], [19], [20] and nematodes lacking either the *pha-4* [21] or *hsf-1* [22] transcription factors. Thus, genotype clearly plays an important role in determining how individual organisms respond to DR.

In the nematode *Caenorhabditis elegans* DR can be modeled both genetically and environmentally, and several different protocols have been described to extend life span via DR [23], [24]. Mutations that reduce food intake by decreasing pharyngeal pumping, such as loss of function alleles of *eat-2*, increase life span and are considered genetic forms of dietary restriction [25]. Environmental models of dietary restriction involve reducing the availability of the bacterial food source for animals cultured either in liquid or solid media. On solid media, extension of life span is observed with complete removal of food during early adulthood, a process termed bacterial deprivation (BD) [26], [27], [28], [29]. Combining mutation of *eat-2* with BD does not result in an additive increase in life span [26], [27], consistent with the model that mutation of *eat-2* and BD act via similar downstream mechanisms to increase life span in *C. elegans*.

The IIS pathway has also been shown in numerous studies to regulate life span in *C. elegans* [30]. Mutations that reduce signaling through this pathway, such as loss of function alleles of the insulin-like receptor *daf-2*, lead to activation of the FOXO-family transcription factor DAF-16 [31], [32]. Longevity-enhancing interventions within this pathway fail to extend life span in animals lacking functional DAF-16. DR is generally accepted to act by mechanisms distinct from the IIS pathway because, with one exception, different DR methods in *C. elegans* extend life span in a DAF-16-independent manner [33], [34]. Genes regulated by DAF-16 enhance resistance to different forms of stress and include superoxide dismutase enzymes, heat shock proteins, cytochrome p450s, UDP-glucuronosyltransferases (UGTs), short-chain dehydrogenase/reductases (SDRs), and glutathione-S-transferases [35], [36]. The abundance of genes involved in detoxification that are regulated by DAF-16 has led to the hypothesis that one component of IIS-mediated longevity is enhanced detoxification [37].

In addition to the positive effects of DR on longevity, several studies in rodents have examined the effects of DR or short-term starvation on resistance to environmental toxins [38], [39], [40], [41], [42], [43], [44], [45], [46], [47], [48], [49], [50]. One particularly interesting recent report suggests that short-term fasting is sufficient to confer striking resistance to a lethal dose of the chemotherapy drug etoposide [51]. The outcomes of these studies have failed to yield a coherent picture, however, with some reporting that DR confers increased resistance to toxicity, but others reporting enhanced sensitivity [39], [40], [46]. Interpretation of these results is further complicated by the fact these studies generally fail to examine life span in the untreated control animals, likely due to the costly and time-consuming nature of longevity studies in rodents.

**Figure 1:**
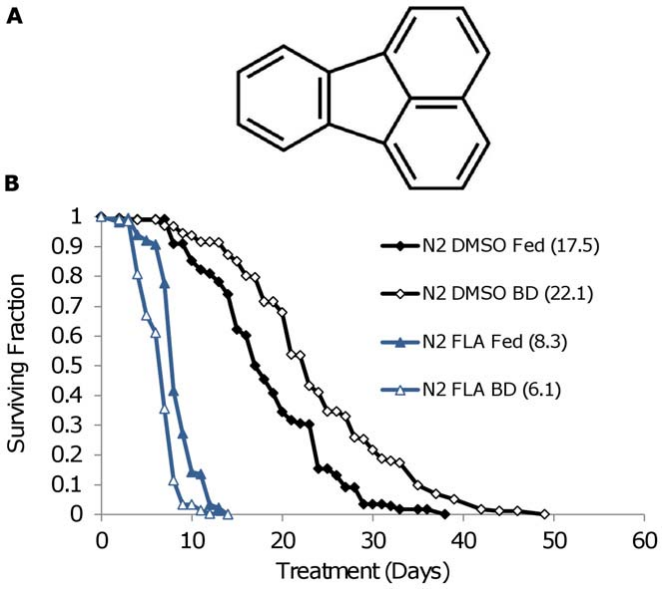
Dietary Restriction Shortens the Life Span of Fluoranthene Treated N2 Adult Animals. (**A**) The chemical structure of fluoranthene (FLA). (**B**) Life span of N2 animals treated continuously with DMSO or FLA starting at adult day 4. Pooled data is shown, mean life span is shown in parentheses. Treatment with FLA shortened life span of N2 animals under control-fed conditions. Bacterial deprivation (BD) further shortened the life span of N2 animals compared to the control-fed. Summary data and statistics for both pooled and individual experiments are provided in **Table S1**.

*C. elegans* offers a potentially useful model system for exploring the effects of DR on resistance or sensitivity to a broad range of environmental toxins. In this study, we examined the impact of two different DR regimens, mutation of *eat-2* and BD, on toxicity of the polycyclic aromatic hydrocarbon (PAH) fluoranthene (FLA) (**Figure 1A**). PAHs are carcinogenic byproducts of combustion which are commonly found near landfills and manufacturing plants [52]. The ability of these compounds to persist in groundwater, soil, and sediment can result in long-term exposure. A recent study reported that FLA is present in soil in Denmark at concentrations between 0.2 and 2 mg/kg [53]. In this study we have examined the effect of 5 mg/kg FLA in the growth medium on survival of *C. elegans* fed a control diet or subjected to DR. We observed that DR induces sensitivity to FLA, and this sensitivity is dependent on the MDT-15 subunit of mediator, a highly conserved complex that regulates transcription through physical interaction with RNA polymerase II [54], [55]. Mediator has been shown to regulate the expression of enzymes involved in xenotoxic response and fatty acid metabolism. Mutation of *mdt-15* reduces basal expression of target genes and prevents their up-regulation in response to environmental changes [56].

## Methods

*Caenorhabditis elegans* were maintained on Nematode Growth Media (NGM) at 20°C with the exceptions of *daf-2 (e1370)* and *daf-16 (mu86)* strains, which were maintained at 15°C. Worms were fed UV-killed *E. coli* OP50 unless otherwise indicated [57]. N2, DA1116 *eat-2 (ad1116)*, CB1370 *daf-2(e1370)*, CF1038 *daf-16(mu86)*, XA7702 *mdt-15(tm2182)* and TJ356 (DAF-16::GFP) [58] worms were obtained from the Caenorhabditis Genetics Center (CGC).

Synchronized egg layings were used to generate cohorts of animals for life spans, as previously described [57], [59]. For *daf-2* and *daf-16* mutant strains, synchronized egg layings were initiated at 15°C and then transferred to 20°C once animals reached the L3 developmental stage. For all strains, L4 larvae were transferred to plates containing 50 µM 5-fluorodeoxyuridine (FUDR) to prevent egg hatching and 100 µg/ml ampicillin (Amp) to prevent bacterial contamination. Day 4 adults were then transferred to FUDR/Amp plates containing 5 ug/ml FLA or equivalent volume of dimethylsulfoxide (DMSO). Animals subjected to BD were transferred to FUDR/Amp plates lacking bacteria, as previously described [29], [57]. For FLA+BD experiments, FLA was included in the BD plates at the time of transfer. Life span experiments were maintained at 20°C and cohorts were evaluated every 1–3 days using tactile stimulation to verify viability of the animals. Animals lost due to foraging off the surface of the agar plates were not included in the data analysis. Differences in median life span were considered significant at a *P*-value of 0.05 by a Wilcoxon rank-sum test. A summary of all life span data included in this report is provided in **Supplementary Tables S1, S2, S3, and S4**.

To evaluate pharyngeal pumping rate, animals were video recorded using a Canon Powershot S3 IS camera under 102X magnification on an Olympus SZ60 dissection microscope. Animals were observed after 24 hours of treatment with FLA or DMSO under either control fed or BD conditions. Videos were scored by individuals blinded to the genotype of the groups. Data were analyzed using a Student's t-test and differences were considered significant for *p*<0.05.

Nuclear localization of DAF-16 in response to FLA was evaluated using DAF-16::GFP transgenic worms, as previously described [59], [60]. Eggs collected by synchronized egg lay were allowed to hatch on NGM plates seeded with UV-killed OP50. Animals were collected 16–22 hours post egg lay (L1 stage) and placed on DMSO control plates with empty vector (EV) RNAi bacteria (for both the control and heat shock groups), control plates with *daf-2(RNAi)* bacteria, or experimental plates containing 5 ug/ml FLA seeded with EV bacteria. Animals were incubated on the experimental plates for 23–30 hours. Heat-shock treated animals underwent a 37°C incubation for the 2 hours immediately prior to quantification of DAF-16::GFP localization. Animals were immobilized using 1 M sodium azide and immediately photographed using a Zeiss SteREO Lumar V.12 microscope. Analysis of the photos was performed by individuals blinded to the genotype of the group.

## Results

### Bacterial deprivation enhances toxicity of fluoranthene

To determine the effect of DR on FLA toxicity we examined the survival of *C. elegans* treated with 5 ug/ml FLA or equivalent volume of DMSO fed either a control diet or subjected to BD beginning at the 4^th^ day of adulthood. As previously reported for animals maintained on NGM [26], DMSO-treated N2 animals subjected to BD lived significantly longer than control-fed DMSO-treated animals (**Figure 1B**). Treatment with FLA significantly shortened the life spans of both control fed and BD animals compared to the DMSO treated controls. Interestingly, FLA treated animals subjected to BD were significantly shorter-lived than control fed animals treated with FLA. These data demonstrate that FLA is toxic to adult *C. elegans*, and that this toxicity is enhanced by BD.

**Figure 2:**
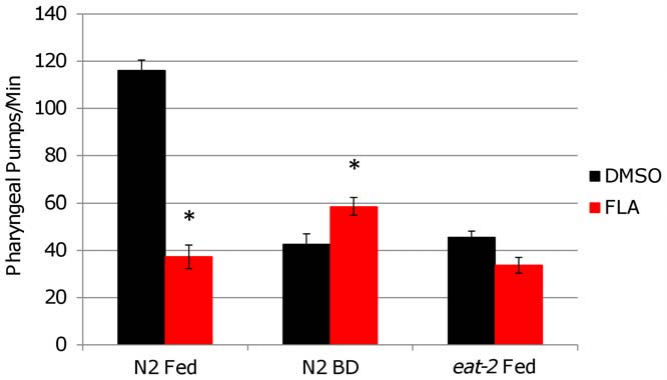
Bacterial Deprivation and Fluoranthene Treatment Reduce Pharyngeal Pumping. Pharyngeal pump rates of N2 and *eat-2(ad1116)* animals after 24 hours treatment with DMSO or FLA under control-fed (Fed) or bacterially deprived (BD) conditions. Pharyngeal pumping was reduced in both FLA treated and BD populations. *denotes p>0.0001 compared to DMSO treated samples. Rates are recorded as pumps per minute. Summary data and statistics are shown in **Table 1**.

We considered the possibility that the enhanced sensitivity to FLA could result from greater uptake of the chemical from the NGM due to elevated rates of pharyngeal pumping in BD animals. Pumping rate was reduced in BD animals under the conditions used here, however, indicating that this is unlikely to be the case (**Figure 2** **,** **Table 1**). Treatment with FLA also significantly reduced pumping in control fed animals. As previously reported [25], *eat-2(ad1116)* animals also showed a reduced pumping rate, relative to N2 animals.

**Table 1:** Summary of Pharyngeal Pumping Data.

Strain	Treatment	N	Mean Pump Rate (pumps/min) +/− SEM	p-value vs DMSO Fed	p-value vs FLA Fed
N2	DMSO Fed	28	116.4+/−4.47	NA	p>0.0001
	DMSO BD	33	42.7+/−4.26	p>0.0001	p = 0.85
	FLA Fed	37	37.3+/−5.01	p>0.0001	NA
	FLA BD	35	58.7+/−3.82	p>0.0001	p>0.0005
*eat-2(ad1116)*	DMSO Fed	9	33.8+/−3.38	NA	p = 0.23
	FLA Fed	8	45.5+/−2.66	p = 0.23	NA

Pharyngeal pumping was observed after 24 hours treatment with FLA or DMSO under control-fed or bacterially deprived conditions. Student's t-test was used to determine statistical significance.

**Table 2:** Summary of DAF-16::GFP Nuclear Localization Data.

	N	Foci positive Animals	% Positive	Mean # foci/animal	p-value
EV	18	1	5.60%	4.2	NA
*daf-2(RNAi)*	35	9	25.70%	5.7	0.035
FLA	40	11	27.50%	11.9	0.018
heat-shock	10	10	100%	115.9	p>0.0001

Animals were scored for presence of nuclear puncta after treatment with DMSO, *daf-2(RNAi)*, FLA or following a 2 hour heat-shock incubation at 37°C. A Student's T-Test was used to determine statistical significance.

**Figure 3:**
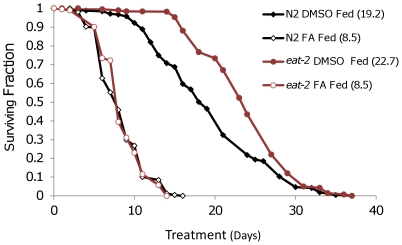
*eat-2(ad1116)* Animals are not Resistant to FLA Treatment. Life spans of N2 and *eat-2(ad1116)* animals after continuous exposure to DMSO or FLA starting at day 4 of adulthood. Pooled data is shown, mean life span is shown in parentheses. Mutation of *eat-2* increased life span in DMSO treated populations compared to N2. FLA treated N2 and *eat-2(ad1116)* populations were not significantly different. Summary data and statistics for both pooled and individual experiments are shown in **Table S2**.

To determine whether enhanced toxicity of FLA results generally from DR or is specific for BD, we examined the effect of FLA on survival of *eat-2(ad1116)* animals. As previously reported on NGM [25], median life span of *eat-2(ad1116)* animals was extended relative to N2 controls on DMSO plates (**Figure 3**). Similar to the case for BD, FLA resulted in a greater proportional shortening of life span in *eat-2(ad1116)* animals relative to N2 animals; however, in this case, there was not a significant difference between N2 and *eat-2* animals treated with FLA. These data indicate that FLA prevents life span extension by two different DR methods, but that only BD results in significantly enhanced toxicity from FLA.

### Reduced insulin/IGF-1-like signaling does not enhance resistance to FLA

**Figure 4:**
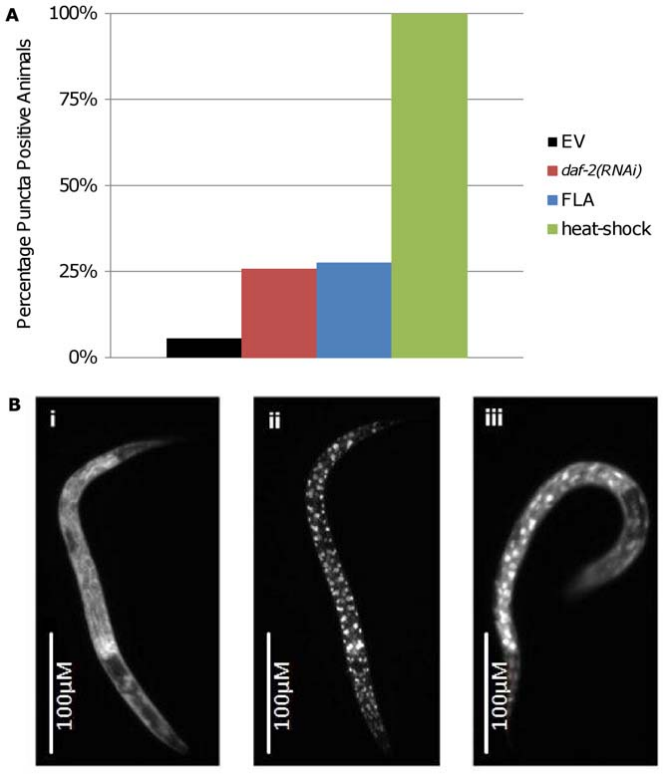
FLA Induces Nuclear Localization of *Daf-16*. Proportion of DAF-16::GFP animals with nuclear puncta after treatment with FLA, DMSO, *daf-2(RNAi)*, or 2 hour incubation at 37°C (A). Treatment with FLA induced nuclear puncta similar to those observed *daf-2(RNAi)* with treatment. (**B**) Images of DAF-16::GFP animals treated with DMSO **(i)**, heat-shock **(ii)**, or FLA **(iii)**. Summary data and statistics are shown in **Table 2**.

DR is thought to modulate longevity in a pathway that is genetically distinct from IIS. Reduced IIS causes DAF-16 to relocalize from the cytoplasm to the nucleus, resulting in increased life span and resistance to different types of stress [58]. To examine whether treatment with FLA results in nuclear localization of DAF-16, we treated animals expressing GFP tagged DAF-16 and followed localization of the tagged protein. After treatment with FLA, 25% of animals displayed nuclear localization of DAF-16, compared to 5% of DMSO treated animals (**Figure 4A–B**). Thus, we conclude that DAF-16 nuclear localization is enhanced by FLA, consistent with activation of DAF-16 in response to FLA treatment.

**Figure 5:**
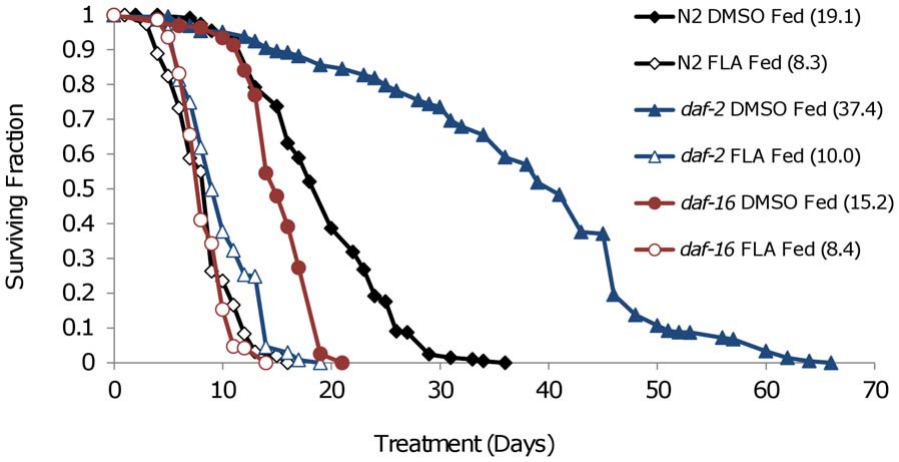
Insulin/IGF-1 signaling does not influence FLA toxicity. Life spans of N2, *daf-2(e1370)*, and *daf-16(mu86)* animals after continuous treatment with DMSO or FLA under control-fed conditions starting at day 4 of adulthood. Mutation of *daf-2* and *daf-16* extended and shortened life span, respectively, compared to N2 in DMSO treated samples. N2, *daf-2(e1370)*, and *daf-16(mu86)* animals displayed significantly shortened life spans when treated with FLA. Summary data and statistics for both pooled and individual experiments are shown in **Table S3**.

To determine whether DAF-16 can promote resistance to FLA, we examined the effect of mutations in *daf-2* and *daf-16* on FLA toxicity. In agreement with previous studies of animals maintained on NGM, control fed DMSO treated *daf-2(e1370)* mutants had an extended life span compared to N2 animals, while *daf-16(mu86)* animals displayed slightly shortened life spans (**Figure 5**). Treatment with FLA shortened the median life spans of both *daf-2(e1370)* and *daf-16(mu86)* animals to 9 and 8 days respectively, suggesting that IIS plays little, if any, role in FLA toxicity.

### MDT-15 promotes FLA toxicity

**Figure 6:**
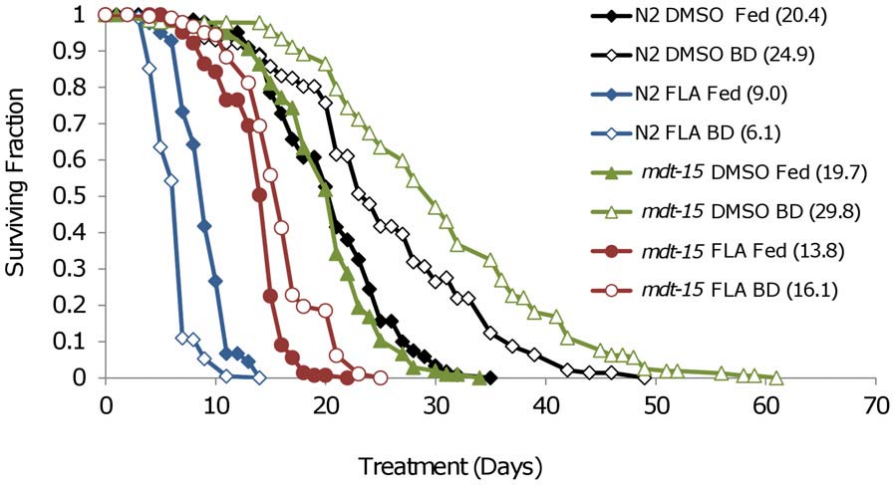
*mdt-15(tm2182)* mutants are resistant to FLA and improve response to BD. Life spans of N2 and *mdt-15(tm2182)* animals after continuous treatment with DMSO or FLA under control fed or BD conditions starting at day 4 of adulthood. *mdt-15(tm2182)* animals had life spans similar to N2 under control fed conditions. Treatment with FLA shortened the life spans of *mdt-15(tm2182)* animals but not as severely as N2 animals. Control fed and BD *mdt-15(tm2182)* animals treated with FLA did not have significantly different life spans. Summary data and statistics for both pooled and individual experiments are shown in **Table S4**.

FLA toxicity requires metabolic activation by cytochrome p450 enzymes [61]. We hypothesized the transcriptional coactivator Mediator may be involved in activation of FLA in *C. elegans*, since it is known to regulate many genes involved in fatty acid metabolism and detoxification, including several cytochrome p450 enzymes [56]. To test this possibility we conducted life spans on *mdt-15(tm2182)* animals, which carry partial deletion allele of the mediator subunit MDT-15. The life spans of *mdt-15(tm2182)* animals did not differ significantly from N2 animals under DMSO control fed conditions, however, deletion of *mdt-15* conferred significant resistance to FLA, compared to N2 animals (**Figure 6**).

In contrast to our results with N2 animals, where BD sensitized animals to FLA toxicity, BD enhanced resistance to FLA in *mdt-15(tm2182)* animals. The survival of *mdt-15(tm2182)* animals in the presence of FLA increased from 13.8 days for control-fed animals to 16.1 days for BD animals. Unexpectedly, *mdt-15(tm2182)* animals in the absence of FLA showed an even more robust life span extension than N2 in response to BD.

## Discussion

Dietary restriction has been proposed as a potential means of increasing health span and life span in people [62], [63]. The importance of genetic and environmental variation on the response to DR is poorly understood, however. As an initial foray into understanding how DR might influence the response to common environmental toxins, we examined the effect of FLA on *Caenorhabditis elegans* under control fed and DR conditions. We anticipated that DR would enhance resistance to FLA, based on the observation that many long-lived mutants are stress-resistant. To our surprise, two different methods of DR failed to enhance resistance to FLA. In fact, BD animals displayed reduced survival in the presence of FLA. Also surprising in light of the fact that DAF-16 localized to the nucleus in response to FLA, we found no evidence that IIS influences FLA toxicity. Mutants with either enhanced or reduced signaling through this pathway showed sensitivity to FLA that was comparable to wild-type N2 animals.

FLA requires metabolic activation for toxicity. Activation is mediated primarily through oxidation by the cyp450 enzymes [52]. Increases in cyp450 enzymes have previously been associated with dietary restriction in mice [38], [64], [65], [66], suggesting that increased activation of FLA may be one potential mechanism for the enhanced toxicity caused by BD in *C. elegans*. These findings are consistent with our observation that mutation of *mdt-15*, which was previously shown to regulate nine different cyp450 enzymes in response to FLA [56], attenuates FLA toxicity in control fed animals and suppresses the enhanced toxicity associated with BD. In fact, BD significantly extends the survival of FLA treated *mdt-15(tm2182)* animals, albeit only to a modest extent. These data support a model whereby activation of FLA requires mediator, and activated FLA is particularly toxic to BD animals. It should be noted that this model does not require enhanced mediator activity in response to DR, only differential sensitivity to the activated toxin.

Although mutation of MDT-15 reduced the sensitivity of animals to FLA, we did not observe a complete resistance to FLA. Several possibilities could account for this observation. First, the *mdt-15(tm2182)* allele is predicted to produce a truncated protein. Therefore, residual MDT-15 activity could promote activation of FLA. Second, not all of the *C. elegans* cyp450 genes are regulated by MDT-15 [56], making it unlikely that cyp450 activity is completely absent in *mdt-15* animals, even if MDT-15 is inactive. Third, although PAH's are thought to be primarily activated by cyp450's, alternative mechanisms of activation have been proposed [67]. Fourth, the native form of FLA may have some inherent toxicity. Platt et al. [68] have reported that FLA induces formation of DNA adducts the absence of metabolic activation under standard laboratory conditions. We also observed that *mdt-15(tm2182)* animals show a slight increase in resistance to FLA under BD conditions. This observation is likely due to up-regulation of additional detoxification pathways that are unrelated to the metabolic activation of FLA.

Our finding that *mdt-15(tm2182)* animals are resistant to FLA differ somewhat from a prior study in which an increased frequency of “scrawny animals” was reported in mediator mutants following 4 days of treatment with FLA [56]. This is likely due to the very different conditions and end points used in the two studies. Under our conditions, we observed a greater than 90% survival for N2 animals at day four of FLA treatment and greater than 99% survival for *mdt-15(tm2182)* mutants. We did not quantify the occurrence of scrawny worms and instead used viability as a measure of toxicity. Our observation that the *mdt-15(tm2182)* allele has relatively little effect on life span under control conditions is notable in light of two studies reporting that RNAi knock-down of *mdt-15* shortens life span [69], [70]. This may indicate that the *tm2182* allele alters MDT-15 activity in a manner that is different from RNAi knock-down. In addition, the role of *mdt-15* as a modulator of longevity may be sensitive to experimental conditions such as food source and temperature. For example, we used killed OP50 *E. coli* as food, whereas the prior reports used live *E. coli*.

The apparent further extension of life span from BD in *mdt-15* mutant animals was unexpected, considering that *mdt-15(tm2182)* animals were neither long nor short-lived under control fed conditions and Rogers et al. [70], observed a reduction in life span extension from mutation of *eat-2* following *mdt-15(RNAi)*. One possibility is that mediator activity is altered by BD in a way that limits the longevity wild-type animals subjected to BD animals. Alternatively, it may be that loss of mediator indirectly affects a process that is important for survival under BD conditions. One interesting possibility is that deletion of mediator alters fat metabolism in a way that allows BD animals to survive in the absence of bacterial food for extended periods of time. It will be of interest to explore this possibility in future studies.

In this study, we have reported that an important environmental toxin, FLA, prevents life span extension from DR or reduced IIS in *C. elegans*. Animals subjected to the extreme form of DR, BD, are more susceptible to FLA, and this enhanced toxicity is suppressed by mutation of the mediator subunit *mdt-15*. We have also shown that *mdt-15* influences the magnitude of life span extension from BD in the absence of FLA. This convincingly demonstrates the point that the effect of DR on life span is robustly influenced by both environmental and genetic components. Although we have no evidence that sensitivity to FLA or other environmental toxins is similarly impacted by caloric intake in humans, it is likely that effects of DR will be strongly influenced by genetic and environmental variation. Understanding the molecular mechanisms that control differential responses to DR in model organisms provides a path toward predicting how such variation will influence the effects of DR and DR mimetics on health and longevity in people.

## Supporting Information

Table S1 **Life span data from N2 animals treated with FLA.** Life span data from individual and pooled experiments. Statistical significance was evaluated by a Wilcoxon Rank-Sum test.
(PDF)

Table S2 **Life span data from** ***eat-2(ad1116)*** **animals treated with FLA.** Life span data from individual and pooled experiments. Statistical significance was evaluated by a Wilcoxon Rank-Sum test.
(PDF)

Table S3 **Life span data from** ***daf-2(e1370)*** **and** ***daf-16(mu86)*** **animals treated with FLA.** Life span data from individual and pooled experiments. Statistical significance was evaluated by a Wilcoxon Rank-Sum test.
(PDF)

Table S4 **Life span data from** ***mdt-15(tm2182)*** **animals treated with FLA.** Life span data from individual and pooled experiments. Statistical significance was evaluated by a Wilcoxon Rank-Sum test.
(PDF)
