# Supplementary material for: The MDT-15 Subunit of Mediator Interacts with Dietary Restriction to Modulate Longevity and Fluoranthene Toxicity in Caenorhabditis elegans
Source: PLoS One. 2011 Nov 21;6(11):e28036. doi: 10.1371/journal.pone.0028036 (PMC3221695; doi:10.1371/journal.pone.0028036)
Supplement: Table S1 — Life span data from N2 animals treated with FLA. Life span data from individual and pooled experiments. Statistical significance was evaluated by a Wilcoxon Rank-Sum test. (PDF) [file pone.0028036.s001.pdf]

Table S1 – Life span data from N2 animals treated with FLA.

Exp #	Strain	Treatment	Median	Mean +/- SEM	p-value (vs DMSO treated)	N
1	N2	DMSO Fed	20	20.05 +/- 0.66	NA	57
		DMSO BD	25	25.57 +/- 0.89	NA	117
		FLA Fed	9	9.00 +/- 0.25	p>0.0001	71
		FLA BD	6	6.48 +/- 0.14	p>0.0001	142
2	N2	DMSO Fed	12	12.20 +/- 0.62	NA	63
		DMSO BD	18	17.30 +/- 0.86	NA	56
		FLA Fed	8	7.03 +/- 0.90	p>0.0001	61
		FLA BD	8	6.82 +/- 0.16	p>0.0001	136
3	N2	DMSO Fed	17	19.03 +/- 0.65	NA	62
		DMSO BD	20	21.00 +/- 1.07	NA	20
		FLA Fed	10	8.83 +/- 0.33	p>0.0001	40
		FLA BD	5	5.32 +/- 0.61	p>0.0001	77
Total	N2	DMSO Fed	17	17.51 +/- 0.46	NA	176
		DMSO BD	21	22.06 +/- 0.69	NA	177
		FLA Fed	8	8.26 +/- 0.17	p>0.0001	184
		FLA BD	6	6.07 +/- 0.08	p>0.0001	359

Life span data from individual and pooled experiments. Statistical significance was evaluated by a Wilcoxon Rank-Sum test.
