# Supplementary material for: The MDT-15 Subunit of Mediator Interacts with Dietary Restriction to Modulate Longevity and Fluoranthene Toxicity in Caenorhabditis elegans
Source: PLoS One. 2011 Nov 21;6(11):e28036. doi: 10.1371/journal.pone.0028036 (PMC3221695; doi:10.1371/journal.pone.0028036)
Supplement: Table S2 — Life span data from eat-2(ad1116) animals treated with FLA. Life span data from individual and pooled experiments. Statistical significance was evaluated by a Wilcoxon Rank-Sum test. (PDF) [file pone.0028036.s002.pdf]

Table S2 – Life span data from *eat-2(ad1116)* animals treated with FLA.

Exp #	Strain	Treatment	Median	Mean +/- SEM	p-value (vs DMSO treated)	N
4	N2	DMSO Fed	18	19.69 +/- 0.97	NA	68
		FLA Fed	8	8.17 +/- 0.32	p>0.0001	76
4	*eat-2(ad1116)*	DMSO Fed	24	23.12 +/- 0.99	NA	33
		FLA Fed	8	8.71 +/- 0.74	p>0.0001	14
5	N2	DMSO Fed	21	21.01 +/- 0.7	NA	82
		FLA Fed	7	8.34 +/- 0.33	p>0.0001	105
5	*eat-2(ad1116)*	DMSO Fed	24	25.42 +/- 0.84	NA	45
		FLA Fed	8	8.14 +/- 0.26	p>0.0001	106
6	N2	DMSO Fed	15.5	16.21 +/- 0.67	NA	64
		FLA Fed	9	7.91 +/- 0.22	p>0.0001	57
6	*eat-2(ad1116)*	DMSO Fed	23	21.06 +/- 0.67	NA	48
		FLA Fed	8	8.66 +/- 0.18	p>0.0001	49
Total	N2	DMSO Fed	18	19.16 +/- 0.52	NA	181
		FLA Fed	8	8.50 +/- 0.18	p>0.0001	237
Total	*eat-2(ad1116)*	DMSO Fed	23	22.72 +/- 0.49	NA	139
		FLA Fed	8	8.51 +/- 0.19	p>0.0001	173

Life span data from individual and pooled experiments. Statistical significance was evaluated by a Wilcoxon Rank-Sum test.
