# Supplementary material for: The MDT-15 Subunit of Mediator Interacts with Dietary Restriction to Modulate Longevity and Fluoranthene Toxicity in Caenorhabditis elegans
Source: PLoS One. 2011 Nov 21;6(11):e28036. doi: 10.1371/journal.pone.0028036 (PMC3221695; doi:10.1371/journal.pone.0028036)
Supplement: Table S3 — Life span data from daf-2(e1370) and daf-16(mu86) animals treated with FLA. Life span data from individual and pooled experiments. Statistical significance was evaluated by a Wilcoxon Rank-Sum test. (PDF) [file pone.0028036.s003.pdf]

Table S3 – Life span data from *daf-2(e1370)* and *daf-16(mu86)* animals treated with FLA.

Exp #	Strain	Treatment	Median	Mean +/- SEM	p-value (vs DMSO treated)	N
7	N2	DMSO Fed	22	21.40 +/- 0.67	NA	61
		FLA Fed	9	9.52 +/- 0.27	p>0.0001	46
7	*daf-2(e1370)*	DMSO Fed	39	36.74 +/- 1.20	NA	79
		FLA Fed	11	10.51 +/- 0.39	p>0.0001	88
7	*daf-16(mu86)*	DMSO Fed	16	11.76 +/- 0.41	NA	67
		FLA Fed	8	9.33 +/- 1.06	p>0.0001	78
8	N2	DMSO Fed	18	17.93 +/- 0.74	NA	64
		FLA Fed	6	6.30 +/- 0.28	p>0.0001	73
8	*daf-2(e1370)*	DMSO Fed	36	33.58 +/- 1.65	NA	51
		FLA Fed	9	8.39 +/- 0.25	p>0.0001	75
8	*daf-16(mu86)*	DMSO Fed	15	14.97 +/- 0.50	NA	48
		FLA Fed	8	7.24 +/- 0.21	p>0.0001	54
9	N2	DMSO Fed	16	18.22 +/- 0.61	NA	78
		FLA Fed	9	9.43 +/- 0.43	p>0.0001	56
9	*daf-2(e1370)*	DMSO Fed	46	40.38 +/- 1.70	NA	77
		FLA Fed	10	10.98 +/- 0.38	p>0.0001	79
9	*daf-16(mu86)*	DMSO Fed	14	15.25 +/- 0.33	NA	78
		FLA Fed	10	8.34 +/- 0.94	p>0.0001	78
Total	N2	DMSO Fed	18	19.12 +/- 0.4	NA	203
		FLA Fed	9	8.31 +/- 0.22	p>0.0001	175
Total	*daf-2(e1370)*	DMSO Fed	41	37.41 +/- 0.98	NA	207
		FLA Fed	9	10.00 +/- 0.22	p>0.0001	242
Total	*daf-16(mu86)*	DMSO Fed	15	15.22 +/- 0.23	NA	193
		FLA Fed	8	8.43 +/- 0.15	p>0.0001	210

Life span data from individual and pooled experiments. Statistical significance was evaluated by a Wilcoxon Rank-Sum test.
