# Supplementary material for: The MDT-15 Subunit of Mediator Interacts with Dietary Restriction to Modulate Longevity and Fluoranthene Toxicity in Caenorhabditis elegans
Source: PLoS One. 2011 Nov 21;6(11):e28036. doi: 10.1371/journal.pone.0028036 (PMC3221695; doi:10.1371/journal.pone.0028036)
Supplement: Table S4 — Life span data from mdt-15(tm2182) animals treated with FLA. Life span data from individual and pooled experiments. Statistical significance was evaluated by a Wilcoxon Rank-Sum test. (PDF) [file pone.0028036.s004.pdf]

**Table S4 – Life span data from *mdt-15(tm2182)* animals treated with FLA.**

| Exp # | Strain | Treatment | Median | Mean +/- SEM | p-value (vs DMSO treated) | N |
|---|---|---|---|---|---|---|
| 1 | N2 | DMSO Fed | 20 | 20.05 +/- 0.66 | | 57 |
| | | DMSO BD | 25 | 25.57 +/- 0.89 | | 117 |
| | | FLA Fed | 9 | 9.00 +/- 0.25 | p>0.0001 | 70 |
| | | FLA BD | 6 | 6.48 +/- 0.14 | p>0.0001 | 142 |
| 1 | *mdt-15(tm2183)* | DMSO Fed | 22 | 20.26 +/- 0.53 | | 62 |
| | | DMSO BD | 31 | 31.37 +/- 1.1 | | 107 |
| | | FLA Fed | 15 | 13.63 +/- 0.34 | p>0.0001 | 73 |
| | | FLA BD | 16 | 16.91 +/- 0.36 | p>0.0001 | 119 |
| 10 | N2 | DMSO Fed | 17 | 19.03 +/- 0.65 | NA | 62 |
| | | DMSO BD | 20 | 21.00 +/- 1.07 | NA | 20 |
| | | FLA Fed | 10 | 8.83 +/- 0.33 | p>0.0001 | 40 |
| | | FLA BD | 5 | 5.32 +/- 0.61 | p>0.0001 | 77 |
| 10 | *mdt-15(tm2183)* | DMSO Fed | 20 | 18.8 +/- 0.93 | NA | 41 |
| | | DMSO BD | 27 | 26.91 +/- 1.09 | NA | 58 |
| | | FLA Fed | 15 | 13.89 +/- 0.35 | p>0.0001 | 74 |
| | | FLA BD | 15 | 14.41 +/- 0.37 | p>0.0001 | 58 |
| Total | N2 | DMSO Fed | 20 | 20.39 +/- 0.54 | NA | 119 |
| | | DMSO BD | 23 | 24.87 +/- 0.78 | NA | 137 |
| | | FLA Fed | 9 | 8.94 +/- 0.22 | p>0.0001 | 90 |
| | | FLA BD | 6 | 6.08 +/- 0.10 | p>0.0001 | 219 |
| Total | *mdt-15(tm2183)* | DMSO Fed | 20.5 | 19.69 +/- 0.49 | NA | 103 |
| | | DMSO BD | 30 | 29.81 +/- 0.84 | NA | 165 |
| | | FLA Fed | 15 | 13.76 +/- 0.25 | p>0.0001 | 141 |
| | | FLA BD | 16 | 16.10 +/- 0.28 | p>0.0001 | 177 |

Life span data from individual and pooled experiments. Statistical significance was evaluated by a Wilcoxon Rank-Sum test.
